# Anaplastic Large Cell Lymphoma Masquerading as Submandibular Sialolithiasis

**DOI:** 10.7759/cureus.11890

**Published:** 2020-12-04

**Authors:** Pramuditha Rajapakse

**Affiliations:** 1 Internal Medicine, Danbury Hospital, Yale School of Medicine, Danbury, USA

**Keywords:** sialolithiasis, non hodgkin lymphoma, cd30+ anaplastic large cell lymphoma, acute spontaneous tumor lysis syndrome, non hodgkin's lymphoma, sialadenitis, anaplastic lymphoma kinase

## Abstract

Anaplastic large cell lymphoma (ALCL) is an aggressive neoplasm of T- or null cell phenotype. It is rarely associated with a leukemic phase. Based on a structured literature review, salivary duct obstruction and sialolithiasis have not been reported as a presenting feature of lymphoma. Here, we report a case of a 74-year-old male who was initially referred to an otolaryngologist for sialolithiasis and was later found to have a rare and aggressive form of lymphoma on further evaluation. He was diagnosed with anaplastic lymphoma kinase (ALK) positive anaplastic large cell lymphoma with a leukemic phase complicated by multiorgan failure and tumor lysis syndrome, leading to death prior to the initiation of chemotherapy. The aim of this report is to make clinicians aware of this unusual presentation, as early recognition and timely referral to the appropriate specialist is important to prevent adverse outcomes. This also emphasizes the importance of exploring the underlying etiology of disease in addition to treating the disease itself, as presenting symptoms can mask the underlying etiology.

## Introduction

The clinical presentation of non-Hodgkin lymphoma (NHL) varies by the subtype of lymphoma and areas of involvement. Common presentations include lymphadenopathy, hepatosplenomegaly, fever, weight loss, and night sweats. Lymphadenopathy can cause obstruction of adjacent structures, resulting in atypical presentations. Based on a structured literature review, salivary duct obstruction and sialolithiasis have not been reported as a presenting feature of lymphoma. The aim of our report is to make clinicians aware of this unusual presentation, as early recognition and timely referral to the appropriate specialist is imperative to prevent the complications of aggressive lymphomas. We would like to emphasize the importance of exploring the underlying etiology of the disease in addition to treating the disease itself, as atypical symptoms can mask the underlying malignancy, leading to delayed diagnosis.

## Case presentation

**Figure 1:**
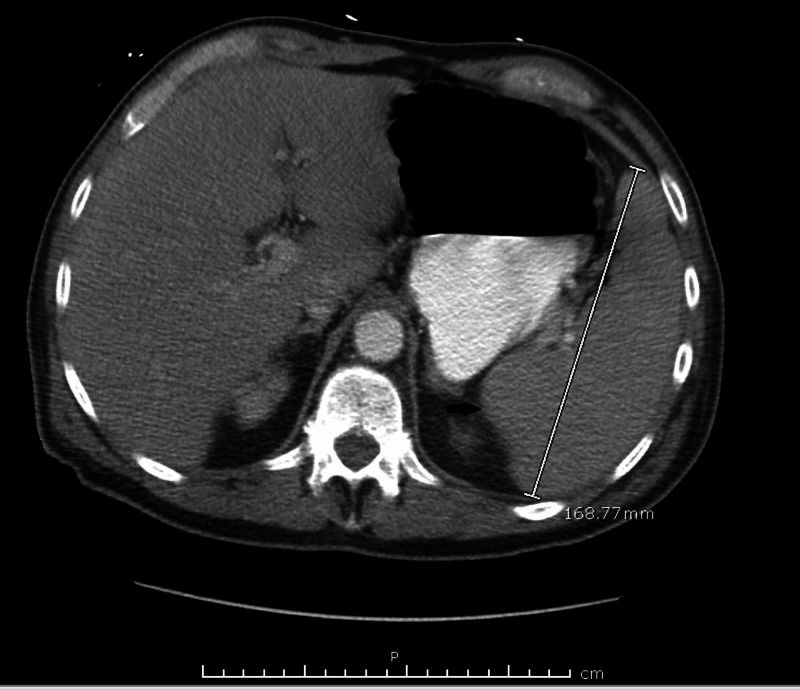
Splenomegaly measuring 16.87 cm

**Figure 2:**
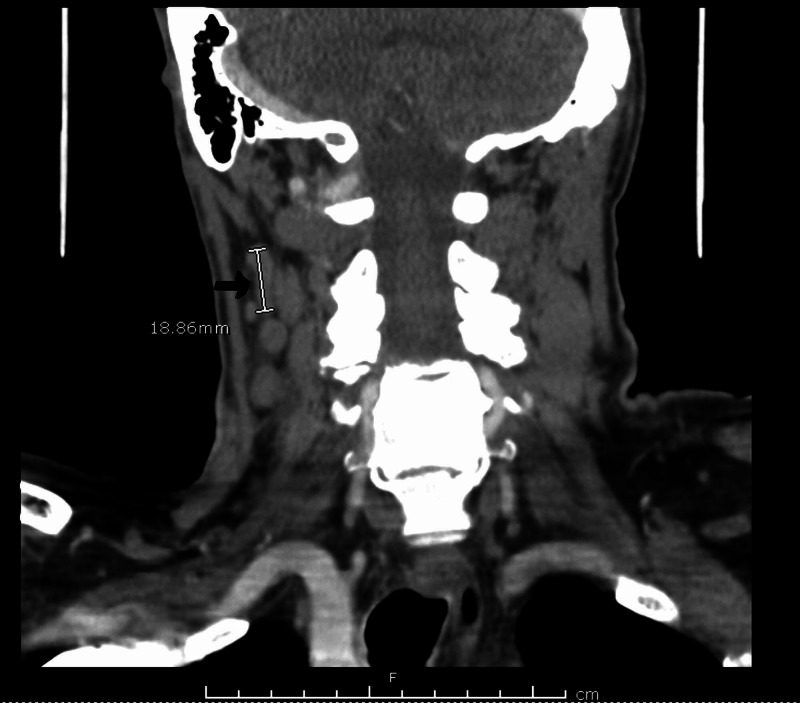
CT neck showing right cervical lymph node measuring 18.86 mm from which biopsy was obtained CT: computed tomography

This is a case of a 74-year-old male presented to our hospital with a one-week history of pain underneath the right jaw before and during meals. A week earlier, he had developed a sudden onset of swelling and pain underneath the right jaw associated with eating and the anticipation of eating. He did not have any constitutional symptoms such as fatigue, anorexia, weight loss, and fever. He was seen by the primary care physician. During the physical exam, it was noted that he had developed right submandibular salivary gland enlargement and was referred to an otorhinolaryngologist, who observed a stone at the opening of the salivary gland duct and cervical lymphadenopathy, as well as submandibular gland enlargement, on examination. A clinical diagnosis of sialolithiasis complicated by sialadenitis was made initially. It was presumed that cervical lymphadenopathy was a result of sialadenitis. At that time, imaging was not performed, as there were no symptoms concerning salivary gland tumor or abscess. He was prescribed cephalexin 500 mg twice daily for five days, which he took with some relief of pain. However, three days later, the patient presented to the emergency department with generalized weakness and fatigue. On examination, he was found to have a firm, palpable right submandibular salivary gland and bilateral cervical lymphadenopathy, right greater than left. Labs were ordered, which revealed significant leukocytosis of 47 x 10^9^ cells/L (range 3.5 x 10^9^ -10 x 10^9^ cells/L). Peripheral blood smear (PBS) was negative for leukemic blasts. A computed tomography (CT) scan with contrast showed multiple enlarged cervical lymph nodes, the largest measuring 18.86 mm (Figure 1) and moderate splenomegaly of 168.7 mm. (Figure 2)

Flow cytometry of peripheral blood was performed. The lymphocytes were mostly T cells accounting for approximately 30% of total cellularity with the expression of cluster of differentiation 3 (CD3). These T cells showed a spectrum of expression of CD3 and CD5, where most of the cells were CD3 and 5+, with a small subpopulation showing lesser expression/loss of CD5 and CD3. All T cells expressed CD7 and CD2 and a majority of these cells showed aberrant expression of CD25. Only rare CD4 positive cells were seen (0.1%). CD8+ cells accounted for approximately 16%. The T cells appeared to partially express TCR alpha-beta (small subpopulation) and were negative for TCR gamma delta. They were negative for CD4, CD16, CD56, CD57, cTdT, CD34, and CD10. Thus, there was an abnormal population of CD8 positive T cells. The differential diagnosis of the peripheral blood smear flow cytometry included a T-cell lymphoproliferative disorder and a reactive process characterized by an expansion of a normal T-cell subset. The patient underwent a right submandibular excisional lymph node biopsy. Two days after admission, the patient was found to be encephalopathic. At this time, labs revealed an elevated uric acid level at 9.6 mg/dL (range 3.7-8 mg/dL), decreased ionized calcium level at 1.04 mmol/ L (range 1.17-1.33 mmol/L), elevated phosphorus level at 5 mg/dL (range: 2.6-4.7 mg/dL), elevated potassium at 5.5 mmol/L (range: 3.5-5.3 mmol/L), elevated blood urea nitrogen (BUN) at 110 mg/dL (range: 6-23 mg/dL), creatinine at 3.1 mg/dL (range: 0.67-1.23 mg/dL), which indicated spontaneous tumor lysis syndrome, complicated by acute kidney injury. The patient required daily hemodialysis. His hospital course was also complicated by new-onset atrial fibrillation and mild pericardial effusion. White blood count continued to increase up to 165 x 10^9^ cells/L (range 3.5 x 10^9^ - 10 x 10^9^ cells/L) of which 33.9% were lymphocytes, and, therefore, was started on high-dose steroids (intravenous methylprednisolone 60 mg every 12 hours) and hydroxyurea 1000 mg every 12 hours for cytoreduction. Hemodialysis was continued daily. The patient continued to deteriorate despite supportive measures and was found to be in acute liver failure, leading to severe conjugated hyperbilirubinemia with a total bilirubin of 7.3 mg/dL (range: 0.0-1.2 mg/dL) and direct bilirubin of 7.2 mg/dL (range: 0.0-0.3 mg/dL). The submandibular lymph node biopsy revealed ALK-positive anaplastic large cell lymphoma (ALK-positive ALCL) with the expression of CD30. The specimen was sent to Yale University, and it was forwarded to Mayo Clinic for detailed expert consultation. The lymph node architecture was partly effaced by an abnormal lymphocyte population growing in the interfollicular zones. They were large with rounded and lobulated nuclear contours, dispersed chromatin, distinct nucleoli, and pale cytoplasm. The abnormal large cells uniformly expressed CD30 and ALK1. t(2;5) was not identified. The treatment options were discussed with the family, which included combination chemotherapy with the addition of the new agent brentuximab vedotin and allogeneic stem cell transplant for consolidation. Unfortunately, the patient remained critically ill from multiorgan failure secondary to spontaneous tumor lysis and thus was a poor candidate for chemotherapeutic intervention. On the twelfth day after admission to the hospital and within three weeks from the onset of symptoms, the patient expired from multiorgan failure secondary to the rapid progression of aggressive disease and spontaneous tumor lysis.

## Discussion

Highly aggressive lymphomas commonly present subacutely or acutely with a rapidly growing mass, constitutional symptoms of fever, night sweats, or weight loss and/or tumor lysis syndrome. In contrast, indolent lymphomas are often insidious, presenting with slowly growing or waxing and waning lymphadenopathy over months or years, hepatomegaly, splenomegaly, and/or cytopenias. A minority of patients initially present with extranodal lymphoma with the involvement of the central nervous system, gastrointestinal tract, or skin. Other presentations include the incidental finding of abnormal laboratory results, oncologic emergencies, and paraneoplastic syndrome. Based on a structured literature review, sialolithiasis or sialadenitis has not been reported as a presenting feature of non-Hodgkin lymphoma except for primary salivary gland lymphoma. Primary non-Hodgkin's lymphoma of the salivary gland is a rare tumor that usually occurs in the parotid gland [1]. They are usually B cell neoplasms associated with Sjögren's syndrome [2]. This patient did not show signs and symptoms of Sjögren's syndrome, and the biopsy was consistent with T cell neoplasm, indicating that his sialolithiasis is unrelated to a primary salivary gland pathology. Anaplastic large cell lymphoma (ALCL) is a lymphoid neoplasm of T or null cell origin and a form of peripheral T cell lymphoma [3]. The majority of patients present with painless lymphadenopathy and are found to have a widespread disease on staging. ALCL often follows a clinically aggressive course. When compared with ALK-negative ALCL, ALK-positive ALCL is usually more responsive to therapy and is associated with a better prognosis. This prognostic advantage is present in both children and younger adults with ALCL, although it has been questioned in older adults as in our patient [4]. He had a very poor prognosis despite being ALK-positive, and he developed complications rapidly prior to initiating chemotherapy. This patient developed a leukemic phase of the disease, which is a rare occurrence in ALCL. Upon literature review, 22 well-documented cases of ALCL in the leukemic phase have been reported previously. The leukemic phase occurs almost exclusively in patients with ALK+ ALCL, similar to the present case. Patients with a leukemic phase of ALK+ ALCL appear to have a poorer prognosis than most patients with ALK+ ALCL [5].

## Conclusions

The aim of our report is to make clinicians aware of this unusual presentation, as early recognition and timely referral to the appropriate specialist is imperative to prevent the complications of aggressive lymphomas. This report highlights the significance of elucidating the etiology of ALK-positive ALCL and the potential treatment course of the disease, as such malignancies could present with varying symptoms likely to mask early diagnosis and appropriate management.
